# Exploring the Relationship Between Mental Health and Early Retirement in U.S. Older Adults: A Gendered Perspective

**DOI:** 10.1093/geroni/igaf122.3050

**Published:** 2025-12-31

**Authors:** Josephine Boateng, Eric Frimpong, Seokmin Kim, Jeffrey Burr

**Affiliations:** University of Massachusetts Boston, Dorchester, Massachusetts, United States; University of Massachusetts Boston, Boston, Massachusetts, United States; University of Massachusetts Boston, Quincy, Massachusetts, United States; University of Massachusetts Boston, Boston, Massachusetts, United States

## Abstract

Aging populations present significant challenges for workforce retention. However, limited research has investigated how mental health, especially depression, is associated with early retirement decisions. This study examined whether depression is associated with early retirement among older adults aged 51-64. We also explored whether there are gender differences in this relationship using longitudinal data from the 2020 and 2022 Health and Retirement Study (N = 4,915). For a sample of persons engaged in paid work in 2020, employment status in 2022 was measured as retired, engaged in paid work, and “other” (e.g., disabled, not in labor force). Multinomial logistic regression models are employed to investigate these research questions. Depression is measured in 2020 with CESD depressive symptoms scale (range 0-8). Descriptive characteristics showed that 52% of respondents were engaged in paid work in 2022. In 2020, the mean age of the sample was 59 years, 60.2% were females, 65% were married, 40% had a college degree or more, and 73% had self-reported good health. Regression results showed that as depression symptoms increased, the likelihood of engaging in paid work in 2022 decreased as compared to being retired (RRR = 0.9467, p = 0.029), suggesting respondents in 2020 with depressive symptoms were more likely to retire two years later. Finally, the relationship between depression and early retirement was statistically significant for men but there was no corresponding effect for women. Study findings provided insights into how mental health challenges are associated with labor force retention informing policies aimed at supporting older workers.

